# Late-Onset Mania With Psychotic Features Precipitated by Umrah Pilgrimage: A Place- and Culture-Associated Psychiatric Syndrome

**DOI:** 10.7759/cureus.112335

**Published:** 2026-07-09

**Authors:** Mohammed H Bogari, Wejdan A Hariri, Abdulrahman F Gassem

**Affiliations:** 1 Psychiatry, Al-Noor Specialist Hospital, Makkah, SAU; 2 Psychiatry, King Abdulaziz Hospital, Makkah, SAU

**Keywords:** case report, culture-associated psychiatric syndrome, late-onset mania, pilgrimage, religion-associated mania, umrah

## Abstract

Place- and culture-associated psychiatric syndromes describe acute psychiatric presentations precipitated by emotionally and spiritually intense experiences in culturally significant settings. We report the case of a 59-year-old widowed Tajik woman with no prior psychiatric history who developed an acute manic episode with psychotic features during her first Umrah pilgrimage to Makkah. The episode was preceded by approximately five days of prodromal decreased sleep, irritability, and repetitive ritualistic behaviors, progressing to elevated and labile mood, pressured speech, flight of ideas, severe behavioral disinhibition, grandiose religious delusions (including the belief that she was a prophet), and persecutory delusions. She required emergency psychiatric admission and rapid tranquillization with intramuscular haloperidol 5 mg and intramuscular lorazepam 2 mg. A comprehensive medical workup, including complete blood count, electrolytes, renal and liver function tests, thyroid profile, inflammatory markers, electrocardiography, and neuroimaging, was unremarkable. She achieved acute clinical stabilization within approximately 40 hours, regained insight, and expressed remorse for her behavior. Her presentation fulfilled DSM-5-TR criteria for a manic episode with mood-incongruent psychotic features, consistent with a diagnosis of Bipolar I Disorder, current episode manic, severe. The duration criterion was satisfied by the need for hospitalization. Differential diagnoses, including delirium, brief psychotic disorder, substance- or medication-induced disorder, and secondary (organic) mania, were systematically excluded. This case illustrates how the convergence of acute physiological stressors (sleep deprivation and circadian disruption), heightened religious salience, and a layered vulnerability profile (late-life onset, positive family psychiatric history, and long-standing unresolved psychosocial trauma) may precipitate acute manic episodes in pilgrimage settings. It is hypothesis-generating and highlights a possible association between pilgrimage-related stressors and psychiatric decompensation in susceptible individuals, warranting systematic study.

## Introduction

Makkah is one of the most spiritually significant locations for Muslims and receives millions of pilgrims annually during Hajj and Umrah [1]. Religious pilgrimages such as Umrah represent profound spiritual and emotional experiences that often involve physical exhaustion, sleep deprivation, disruption of circadian rhythms, crowd-related stress, and heightened spiritual arousal. Although generally beneficial, such environments may precipitate acute psychiatric decompensation in susceptible individuals [2].

Several place- and culture-associated psychiatric syndromes have been described, including Jerusalem syndrome, Hajj-related psychosis, Stendhal syndrome, and Paris syndrome [3-6]. To the best of our knowledge, however, this is the first published report describing a late-onset manic episode occurring during Umrah in Makkah.

A manic episode is characterized by a sustained period of abnormally elevated, expansive, or irritable mood and increased goal-directed activity or energy, accompanied by features such as inflated self-esteem or grandiosity, reduced need for sleep, pressured speech, flight of ideas, distractibility, and excessive involvement in activities with high potential for harmful consequences [7]. Untreated, manic episodes typically persist for several months. Pressured speech and increased verbal output are the most common features (approximately 90-98%), followed by psychomotor activation (~87%) and reduced sleep need (~81%); hypersexuality (~57%) and excessive spending (~55%) are less common but characteristic. Psychotic features such as grandiose or persecutory delusions may occur in more severe episodes lasting at least one week or requiring hospitalization [8].

First-onset mania after the age of 50, defined here as late-onset mania, accounts for approximately 5-10% of bipolar admissions among older adults and carries a higher likelihood of secondary etiologies, including cerebrovascular disease, neurodegenerative disorders, metabolic derangements, endocrinopathies, autoimmune encephalitis, and substance- or medication-induced states [9]. A comprehensive medical workup is therefore essential before attributing a new manic episode to a primary mood disorder, particularly in the absence of a premorbid psychiatric history. Environmental and psychosocial precipitants, including sleep deprivation, circadian disruption, intense emotional arousal, and culturally significant stressors, are well-recognized triggers of affective decompensation in vulnerable individuals [10].

The interaction between these biological vulnerabilities and contextual stressors is especially relevant in pilgrimage settings such as Hajj and Umrah, where physical, emotional, and spiritual demands are concentrated within a brief and highly immersive period. Late-onset first-episode mania is uncommon and may phenomenologically overlap with brief psychotic disorder and acute and transient psychotic disorder when the episode is short-lived and fully remits.

In this context, we present the case of a 59-year-old woman with no prior psychiatric history who developed an acute manic episode with psychotic features during her first Umrah, contributing to the limited but growing literature on pilgrimage-related affective psychosis.

The objectives of this report are threefold: to describe the clinical presentation of a late-onset first-episode manic episode with psychotic features arising during Umrah; to detail the diagnostic reasoning and systematic exclusion of alternative etiologies; and to discuss potential pilgrimage-related precipitants and their relevance to culturally informed assessment.

## Case presentation

Patient demographics and background

The patient was a 59-year-old widowed Tajik woman, mother of five children, who presented to the emergency department in Makkah during her first Umrah pilgrimage. She was born and raised in a rural village in Tajikistan, completed Quran school, and had no formal secular education beyond this. She managed her household independently and performed all daily domestic tasks without assistance. She spoke limited Arabic, and all clinical assessments were conducted with the assistance of a qualified translator.

Her medical history was notable only for chronic, untreated arterial hypertension. She had no prior neurological, psychiatric, or substance-use history. This history was established through patient and family report; no prior medical or psychiatric records were available for review, as the patient was a foreign national in Makkah solely for Umrah, and the reliance on collateral history is acknowledged as a limitation.

She had never previously travelled outside Tajikistan, and the present journey to Makkah represented both her first international travel and the realization of a lifelong spiritual aspiration.

Premorbid psychosocial history

The patient was widowed approximately 10 years prior to presentation. Her marriage, which lasted several decades, was characterized by chronic intimate-partner violence: her husband was reported by family members to have been verbally and physically abusive throughout the marriage, with frequent episodes of yelling, beating, and emotional degradation. Despite these prolonged and severe stressors, she did not disclose her suffering, did not seek psychiatric or social support, and continued to fulfil all maternal and household responsibilities.

She raised her five children largely alone, both during the marriage and after her husband's death. She had not previously experienced any episode of pathological elevated, depressed, or psychotic mood, and had not exhibited behavioral disturbance during these earlier and arguably more severe psychosocial adversities. Family members described her premorbid personality as "calm, loving, devoted, and patient," but consistently noted that she "never speaks about her feelings to anyone" - a long-standing, culturally shaped pattern of emotional suppression and stoic coping.

This premorbid profile is clinically significant: it establishes that severe and chronic psychosocial trauma, including domestic violence, widowhood, and the burden of single parenthood, did not, in isolation, precipitate any affective or psychotic decompensation across decades. The first such episode occurred specifically in the context of pilgrimage, in association with acute physiological stressors and heightened spiritual salience, suggesting that pilgrimage-related factors represented a qualitatively distinct precipitant acting on a layered vulnerability substrate.

Family psychiatric history

Family history was notable for maternal-line psychiatric vulnerability. The patient's mother was reported by family members to have experienced recurrent episodic disturbances of mood and behavior resembling those observed in the patient, although these were never formally diagnosed or treated, and no medical records were available. Although these episodes could not be characterised in terms of symptom profile, duration, or frequency, a first-degree family history of severe mood-behavioural disturbance is diagnostically relevant, representing a heritable loading that raises the prior probability of a primary mood disorder and supports the diagnosis of bipolar I disorder over secondary or organic aetiologies. No other first- or second-degree relatives were reported to have psychiatric illness, suicide, or substance-use disorders.

Pilgrimage context and prodrome

The patient is a devout Sunni Muslim from her home village in Tajikistan, where she observed traditional Hanafi-influenced Islamic practice consistent with the religious culture of Central Asia. She prayed regularly throughout her adult life, and family members described her premorbid religious practice as devout but measured, without ritual scrupulosity or excessive religiosity [11]. She had described Umrah to her family for many years as her "greatest dream," and the present pilgrimage represented its long-awaited realization.

The patient travelled to Saudi Arabia, accompanied by adult family members. She received no formal pre-departure psychological or psychiatric screening. Pre-departure preparation was emotionally intense: she reportedly fasted intermittently, slept poorly in the days before travel, and was described as exhilarated and tearful upon arrival in Makkah.

After commencing Umrah rituals, she began to repeat the Tawaf and Sa'i sequences several times beyond the ritual requirement, expressing a need to do them perfectly [12]. Approximately five days before her emergency presentation, family members observed subtle but progressive behavioral changes: markedly decreased need for sleep (reportedly less than two hours per night), pressured speech, increased motor activity, irritability, and disinhibition. These prodromal features escalated over several days into an overt manic episode with psychotic features, as directly reported by the patient and corroborated by her family.

Acute presentation

At presentation, vital signs were within normal limits apart from mild hypertension (blood pressure 150/90 mmHg); heart rate, respiratory rate, temperature, and oxygen saturation were normal, with no autonomic instability. The patient exhibited a persistently elevated and irritable mood with affective lability. Speech was pressured, loud, excessive, and difficult to interrupt. She spoke rapidly with frequent topic shifts and bursts of inappropriate laughter.

At one moment, she expressed grandiose religious ideas, stating that "we are all prophets," and within seconds shifted to flirtatious behavior toward the on-call psychiatrist, making an inappropriate complimentary remark about the clinician's appearance - verbal only, consistent with mild behavioural disinhibition rather than overt hypersexuality; she also exhibited flight of ideas and severe behavioural disinhibition. She expressed grandiose religious delusions, repeatedly stating that she was a prophet and that others around her were also prophets. She voiced persecutory delusions, misidentifying hotel guests as non-Muslims who intended her harm and interpreting a minor scratch from a domestic cat as an attack by an evil spirit (jinn).

Her behavior escalated to severe psychomotor agitation, with physical aggression toward family members and damage to hotel property. Hotel staff, alerted by shouting, found the room in considerable disarray with several broken furnishings and contacted the police and the Red Crescent, who facilitated her transfer to the hospital. She was brought to the emergency department by ambulance under restraint. There was no clouding of consciousness, no fluctuation in attention, no autonomic instability, and no focal neurological signs. According to collateral information provided by the family, the patient remained consistently oriented to time, place, and person throughout the course of her illness and hospitalization.

Delirium was actively excluded: the Confusion Assessment Method was negative [13], digit span was intact, and the level of consciousness and attention remained stable without fluctuation across serial examinations (detailed under the subheader "Diagnostic considerations and differential diagnosis").

Her religious beliefs and behaviors during the episode were excessive, idiosyncratic, and inconsistent with her premorbid devout but measured religious practice, as independently confirmed by multiple family members.

Investigations and management

On admission, the patient required immediate rapid tranquillization with intramuscular haloperidol 5 mg and intramuscular lorazepam 2 mg. She was admitted to the inpatient psychiatric service. Investigations included complete blood count, serum electrolytes (including calcium and magnesium), renal and liver function tests, fasting glucose, thyroid-stimulating hormone and free T4, C-reactive protein, erythrocyte sedimentation rate, urinalysis, electrocardiography, and non-contrast computed tomography of the brain. All results were within normal limits. Electroencephalography and urine toxicology were not performed. Collateral history obtained from family members confirmed no use of prescription medications, over-the-counter stimulants, traditional or herbal preparations, or recreational substances; substance-induced mania or psychosis was excluded on the basis of collateral and patient history alone.

She remained in a structured inpatient environment with regular sleep-wake cycles and controlled stimulation and continued haloperidol 5 mg intramuscularly twice daily and clonazepam 2 mg orally twice daily. Symptoms attenuated progressively, and she achieved acute clinical stabilization within 40 hours of admission. Following stabilization, maintenance pharmacotherapy was discussed with the patient and her family in view of the established diagnosis of bipolar I disorder; the family elected to defer initiation of a mood stabiliser until return to their home country, where ongoing psychiatric follow-up could be arranged. She was discharged on oral clonazepam 2 mg once daily to cover the immediate travel period, in a stable euthymic state, with a written clinical summary, treatment recommendations, and structured psychoeducation regarding relapse risk, sleep hygiene, and the importance of long-term follow-up.

Mental status examination at presentation

On structured examination, the patient's appearance was dishevelled under restraint; her behaviour was uncooperative and agitated. Speech was pressured, loud, and difficult to interrupt. Mood was self-reported as elevated; affect showed marked lability oscillating between euphoria and hostility. Thought process was disorganized, with prominent flight of ideas. Thought content included grandiose religious delusions and persecutory delusions, as detailed above. No hallucinations were elicited. Insight was absent, and judgment was severely impaired.

Assessment of orientation was initially challenging due to the patient's pressured speech, frequent laughing episodes, and distractibility. She was clearly oriented to the person; when asked about time and place, she initially responded by repeating the examiner's questions while laughing inappropriately. With repeated redirection, she was eventually able to correctly identify both place and time. There was no observable evidence of clouded consciousness or fluctuating attention.

Mental status examination post-stabilization

Following stabilization, the patient presented as neatly dressed and well-groomed, with calm, cooperative, and engaged behavior and adequate eye contact. Speech was clear and coherent, normal in rate, volume, and amount. Mood was euthymic, and affect was congruent and appropriate. Thought process was logical, coherent, and goal-directed. She denied hallucinations, delusions, or thoughts of self-harm or harm to others. Although a formal standardized cognitive battery (e.g., MMSE or MoCA) was not administered, general cognitive function was intact on clinical examination, with preserved orientation, attention, and memory and no evidence of fluctuating consciousness; insight and judgment were good. She expressed remorse for her behavior during the episode and verbalized a clear understanding that her actions had been out of character. Collateral assessment by family members confirmed a return to her premorbid baseline functioning.

Diagnostic considerations and differential diagnosis

Assessment was conducted by board-certified psychiatrists at King Abdulaziz Hospital, Makkah. Diagnosis was established according to the operational criteria of DSM-5-TR and ICD-11 [14-16]. Mental status examination was performed at presentation and repeated serially following clinical stabilization. Collateral history was obtained independently from accompanying family members through a qualified medical translator.

The patient's presentation fulfilled DSM-5-TR criteria for a manic episode: a distinct period of abnormally and persistently elevated, expansive, and irritable mood with markedly increased goal-directed activity and energy, accompanied by inflated self-esteem and grandiosity, decreased need for sleep, pressured speech, flight of ideas, distractibility, and excessive involvement in activities with high potential for harmful consequences. The DSM-5-TR duration criterion (≥1 week) is explicitly waived when hospitalization is required, as was the case here. Because the episode included both mood-congruent (grandiose religious) and mood-incongruent (persecutory) delusions, the appropriate DSM-5-TR specifier is "with mood-incongruent psychotic features." The full diagnostic label is therefore Bipolar I Disorder, current or most recent episode manic, severe, with mood-incongruent psychotic features [15].

The episode caused marked impairment in social and personal functioning, posed a clear risk to self and others, and required emergency admission and rapid tranquillization. Several alternative diagnoses were systematically considered and excluded:

Delirium

There was no clouding of consciousness, no fluctuating attention, no disorientation, no perceptual disturbance suggestive of an acute confusional state, and no autonomic instability. Family members denied any changes in orientation or periods of inattention on the same day. Laboratory investigations, inflammatory markers, and neuroimaging were unremarkable, which would be atypical for delirium related to an unresolved organic cause. Delirium was formally assessed using the Confusion Assessment Method, which was negative [14]. The digit span was intact, and the level of consciousness and attention remained stable without fluctuation across serial examinations. The orientation difficulty noted initially reflected manic distractibility, pressured speech, and inappropriate laughter rather than a disturbance of consciousness.

Brief Psychotic Disorder (DSM-5-TR) and Acute and Transient Psychotic Disorder (ICD-11 6A23)

The phenomenology shares features with these entities, particularly the acute onset, the presence of an identifiable psychosocial stressor, and the rapid full remission with return to premorbid functioning. However, the clinical picture was dominated by a sustained, mood-congruent manic state - elevated and irritable mood, pressured speech, flight of ideas, decreased need for sleep, and grandiosity - rather than by primary psychotic symptoms with secondary mood change. The psychotic features occurred within the mood episode rather than independently of it. Mental status examination was therefore more consistent with a manic episode than with an acute psychotic episode [15-16].

Substance- or Medication-Induced Disorder

Collateral history excluded all identifiable substance or medication exposures. Although urine toxicology was not performed, the absence of any identifiable exposure and the clinical course argue against a substance-induced aetiology.

Secondary (Organic) Mania

Late-onset first-episode mania mandates exclusion of cerebrovascular disease, frontal lobe pathology, neurodegenerative disease, hyper- or hypothyroidism, hypercalcemia, infections, autoimmune encephalitis, and intracranial mass lesions. In this patient, complete blood count, electrolytes, renal and liver function, thyroid profile, inflammatory markers, and brain neuroimaging were all unremarkable, and there were no focal neurological signs [17].

Cultural Concepts of Distress

The patient's reference to jinn-related causation reflected her cultural framework for interpreting her symptoms; this interpretation was patient-derived and was incorporated into her psychotic content rather than constituting a diagnosis. Cultural formulation must distinguish idioms of distress and cultural explanatory models from primary psychiatric diagnosis.

## Discussion

This case represents an acute, context-dependent manic episode with psychotic features precipitated by intense religious immersion. The phenomenology aligns with pilgrimage-associated psychiatric syndromes and broader place-triggered psychopathology (Table 1).

**Table 1:** Comparison of place- and culture-associated psychiatric syndromes.

Syndrome	Triggering environment	Typical population	Core psychotic features	Relevance to present case	Reference
Hajj-related psychosis	Religious rituals, crowd density, sleep deprivation, and emotional arousal during Hajj or Umrah	Pilgrims, often first-episode cases	Psychosis or mania with religious or culturally contextualized delusions, agitation, behavioural disinhibition	Directly applicable; the present case occurred during Umrah	Awara et al. [4]
Jerusalem syndrome	Exposure to the religious and spiritual intensity of Jerusalem	Pilgrims and tourists, often without prior psychiatric history	Acute psychosis or mania with grandiose religious delusions, identity disturbance, and ritualistic behaviour	Closely parallels this case in terms of religious grandiosity, acute onset, and rapid stabilization	Bar-el et al. [3]
Stendhal syndrome (hyperkulturemia)	Exposure to emotionally overwhelming art or aesthetic environments (e.g. Florence museums)	Tourists, art enthusiasts	Anxiety, depersonalization, derealization, autonomic symptoms, mood elevation, hallucinations, occasional psychosis	Demonstrates how intense emotional or aesthetic overload can precipitate transient psychiatric symptoms	Innocenti et al. [5]
Paris syndrome	Cultural dissonance and unmet idealized expectations during travel to Paris	Most commonly Japanese tourists	Anxiety, paranoia, derealization, depersonalization, mood disturbance, transient psychosis	Illustrates environmental and cultural stress as precipitant, contrasting with the positive spiritual overload in this case	Uemoto et al. [6]
Present case (Umrah-triggered mania)	Intense religious immersion, ritual repetition, sleep deprivation, spiritual arousal	First-episode pilgrim; older adult with latent vulnerability	Acute mania with mood-incongruent psychotic features, religious grandiosity, aggression, disinhibition	Fits within the spectrum of place- and culture-associated psychiatric syndromes	Current study

Similar presentations have been described during religious pilgrimages. Reports of Hajj-related psychosis and Jerusalem syndrome-like episodes emerging in Makkah highlight the role of spiritual intensity and environmental stress in precipitating transient psychosis [3, 4]. Our case parallels the report by Awara et al. [4] in several clinically important respects: both patients were women in their sixth or seventh decade with no prior psychiatric history; both were undertaking pilgrimage to Makkah for the first time; both had medical comorbidity (untreated hypertension in our patient; diabetes and hypertension in Awara’s); both presented with acute decompensation during ritual performance; and in both cases the comprehensive medical workup, including neuroimaging, was unremarkable. The two cases differ in core phenomenology - delusional misidentification with persecutory features in Awara’s case, manic episode with grandiose religious and persecutory delusions in ours - but converge in supporting the existence of a clinically meaningful pattern of late-onset, first-episode psychiatric decompensation in elderly women undertaking pilgrimage to Makkah for the first time.

Other cases reported worldwide demonstrate that exposure to intense emotions and culturally valued ideas about a place can trigger psychiatric manifestations specific to that location [5, 6]. Pilgrimage-specific data, although limited, support the recognition that elderly first-time pilgrims with no prior psychiatric history can experience acute psychiatric decompensation. In one large series, mood disorders accounted for 22% of psychiatric presentations during Hajj, with 52% representing first-episode cases [18].

Late-onset mania, defined as the first manic episode occurring after the age of 50, is more common than is often appreciated. In a systematic review of 18 studies comprising 1,519 elderly psychiatric inpatients, the pooled prevalence of late-life mania was 6.0% (95% CI: 4.8-7.2%); among elderly bipolar inpatients, late-onset mania accounted for a mean of 44.2% of cases (95% CI: 37.3-51.1%), and approximately one-third of elderly manic inpatients were experiencing their first lifetime episode [19].

In older adults, new-onset mania necessitates thorough evaluation to exclude secondary etiologies such as neurological disease, metabolic abnormalities, infections, or autoimmune conditions, In this case, neuroimaging, laboratory results, and cardiovascular evaluation were unremarkable [20]. Although the patient had chronic hypertension, no evidence suggested that it played a direct role in symptom onset. This rapid trajectory of stabilization further supports a stress-induced, context-dependent psychiatric episode acting upon an underlying biological vulnerability rather than a chronic, established mood disorder presenting de novo.

The rapid clinical stabilization observed in our patients within approximately 40 hours of admission warrants specific comment, as this timeframe is shorter than the natural course typically described for primary manic episodes. We interpret this trajectory as reflecting acute symptom suppression secondary to three concurrent interventions rather than spontaneous resolution of the episode itself. First, the pharmacological combination administered - intramuscular haloperidol with intramuscular lorazepam - is among the most rapidly acting interventions available for acute manic agitation, with significant reduction of manic symptoms documented within three hours of administration in controlled trials [21]. Second, the restoration of regulated sleep-wake cycles in the controlled inpatient environment reversed what is recognized as a final common pathway to manic decompensation [22]; given that progressive sleep curtailment had been a prominent feature of her prodrome, the restoration of sleep alone is likely to have contributed substantially to symptom attenuation. Third, admission removed the patient from the immersive pilgrimage environment that had functioned as the active precipitant, paralleling the rapid clinical improvement described for related place- and culture-associated syndromes. Importantly, although the speed of stabilization was unusual, it does not exclude the diagnosis of bipolar I disorder. The rate of treatment response is not a diagnostic criterion in either DSM-5-TR or ICD-11.

We acknowledge, however, that what we describe as clinical stabilization may represent acute symptom suppression rather than full natural-course episode resolution; this distinction is discussed further in the Limitations section.

This episode is best understood as the convergence of multiple precipitants acting on a vulnerable substrate. The pilgrimage represented the realization of a lifelong spiritual aspiration; from her arrival in Makkah, the patient was described as continuously joyful and emotionally activated, with markedly reduced sleep both because she did not feel the need to sleep and because she did not wish to interrupt her devotional activity. Goal-attainment events of high personal significance are recognized triggers of manic decompensation in vulnerable individuals, and sleep deprivation is a well-established final common pathway to mania [23]. In this patient, sustained positive affect appears to have produced progressive sleep curtailment, closing a self-reinforcing loop in which reduced sleep further amplified mood elevation and goal-directed activity. The pilgrimage environment compounded these effects through circadian shift, heat exposure, fasting, and crowd-related arousal.

In this report, vulnerability is defined as a multidimensional construct rather than as any single risk factor. The patient’s vulnerability profile comprised a positive maternal-line family history of episodic mood and behavioural disturbance; advanced age with associated reductions in dopaminergic regulatory capacity; untreated chronic hypertension as a marker of subclinical cerebrovascular risk; a sociolinguistic and cultural transition into a foreign environment with translator-dependent communication; chronic and unprocessed psychosocial trauma (long-standing intimate-partner violence, widowhood, single parenthood); a long-standing alexithymic coping style; and acute physiological precipitants intrinsic to the pilgrimage (sleep deprivation, circadian disruption, crowd exposure, heat). Chronic hypertension alone is not asserted as causal; rather, it represents one node within a layered substrate that interacted with acute precipitants to permit affective decompensation.

Overall, this case underscores the importance of recognizing that culturally and spiritually significant experiences can interact with biological vulnerability to precipitate acute psychiatric symptoms, particularly in older adults. Clinicians working in pilgrimage settings should maintain heightened awareness of such presentations and conduct comprehensive assessments to distinguish primary psychiatric disorders from secondary or situationally triggered conditions.

Limitations

Several limitations should be considered when interpreting this report. First, this is a single-case observation and therefore precludes any causal inference; the proposed mechanisms are hypothesis-generating rather than confirmatory. Second, electroencephalography and MRI were not performed, and although the clinical picture and neuroimaging argued strongly against ictal phenomena, non-convulsive seizure activity cannot be definitively excluded. Non-contrast CT demonstrated no acute intracranial pathology; MRI was not obtained, as it was declined by the on-call radiology service. CT has lower sensitivity than MRI for the subtle structural lesions associated with secondary mania, which is acknowledged as a limitation. Third, urine toxicology was not obtained; substance- or medication-induced etiology was excluded by collateral history alone. Fourth, all clinical assessments were conducted with the assistance of a medical translator; fifth, the follow-up period reported here is limited to the immediate post-stabilization assessment in Makkah; longitudinal outcome data after the patient’s return to Tajikistan are not available, and we cannot exclude future evolution into a recurrent affective disorder.

## Conclusions

Acute manic or psychotic episodes may emerge in previously healthy individuals during intense religious or culturally significant experiences. Clinicians should perform comprehensive medical evaluations, recognize cultural and spiritually relevant triggers, and intervene promptly as required. Although such episodes may resolve rapidly with appropriate intervention, This case is hypothesis-generating and raises the possibility of an association between pilgrimage-related stressors and psychiatric decompensation in susceptible individuals, warranting systematic study, rather than supporting recognition of a distinct syndrome. Culturally informed assessment is essential for accurate diagnosis, management, and prognosis.
